# Association between cadmium exposure and diabetes mellitus risk: a prisma-compliant systematic review and meta-analysis

**DOI:** 10.18632/oncotarget.21991

**Published:** 2017-10-20

**Authors:** Ming Wu, Jukun Song, Chen Zhu, Yadong Wang, Xinhai Yin, Guanglei Huang, Ke Zhao, Jianguo Zhu, Zhuhui Duan, Lingkai Su

**Affiliations:** ^1^ Department of Emergency Medicine, Guizhou Provincial People's Hospital, Guizhou, China; ^2^ Department of Oral and Maxillofacial Surgery, Guizhou Provincial People's Hospital, Guizhou, China; ^3^ Guiyang Hospital of Stomatology, Medical College, Zunyi Medical College, Guiyang, China; ^4^ Department of Urology, Guizhou Provincial People's Hospital, Guizhou, China; ^5^ Affiliated Hospital of Stomatology, Medical College, Zhejiang University, Hangzhou, China

**Keywords:** cadmium, diabetes mellitus

## Abstract

Cadmium (Cd) is a pollutant with multiple adverse health effects: cancer, renal dysfunction, osteoporosis and fracture, and cardiovascular disease. Several population-based studies found an association between Cd and diabetes mellitus (DM), but this association is inconsistent with other research. We conducted meta-analysis to examine relationship between urinary/blood Cd exposure and DM risk. Pertinent studies were identified by searching PubMed and Embase databases, and combined odds ratio (OR) and corresponding 95% confidence interval (CI) were applied to evaluate said association. Meta-analysis showed that high U-Cd exposure is not correlated with DM risk (OR = 1.19; 95% CI = 0.83–1.71), and high B-Cd exposure is also not associated with increased risk of DM (OR = 1.16; 95% CI = 0.84-1.62) in the general population. Subgroup and sensitivity analysis proved similar results, with little evidence of publication bias. This meta-analysis suggests that high U-Cd/B-Cd exposure may not be risk factor for DM in general populations. However, large prospective studies are needed to confirm this finding.

## INTRODUCTION

Burden of diabetes is increasing globally. In 2008, 347 million people worldwide suffered from diabetes, and this number is well above earlier estimations from the World Health Organization [1, 2]. The number of people with diabetes is expected to reach 366 million worldwide in 30 years; therefore, preventative actions are needed to mediate this global issue [3]. Type II diabetes mellitus (T2DM) is primarily characterized by metabolic disorders and abnormally high blood sugar (hyperglycemia) because of low insulin levels with or without abnormal resistance to insulin action; T2DM accounts for 90% of diabetes cases [4, 5]. Hence, novel preventable risk factors must be identified immediately. Established risk factors for T2DM include age, family history, genetic variants, obesity, and physical inactivity [2, 6, 7]. Beyond these established risk factors, unidentified environmental factors may influence development of DM [8–10].

Health problems related to heavy metal exposure caused worldwide concern recently. Cadmium (Cd) is a naturally occurring, non-essential toxic metal and also an industrial and agricultural pollutant [11]. Cd exhibits high rate of soil-to-plant transfer; therefore, general population is primarily exposed to Cd via food ingestion and tobacco smoke inhalation [12]. Observational studies suggest association between body burden of Cd and T2D and/or prediabetes and showed positive [13–21] and null associations [8, 22–31]. These studies used small sample sizes, which possibly hindered detection of correlation. Therefore, public health will be significantly affected by increased body burden of Cd exposure and common incidence of DM and risk factors for its development. Therefore, we systematically performed meta-analysis to evaluate association between urinary/blood Cd exposure and DM risk. Systematic search and review processes were conducted according to the Preferred Reporting Items for Systematic Reviews and Meta-Analyses Statement criteria [32].

## RESULTS

### Literature search, study characteristics, and quality

**Figure 1:**
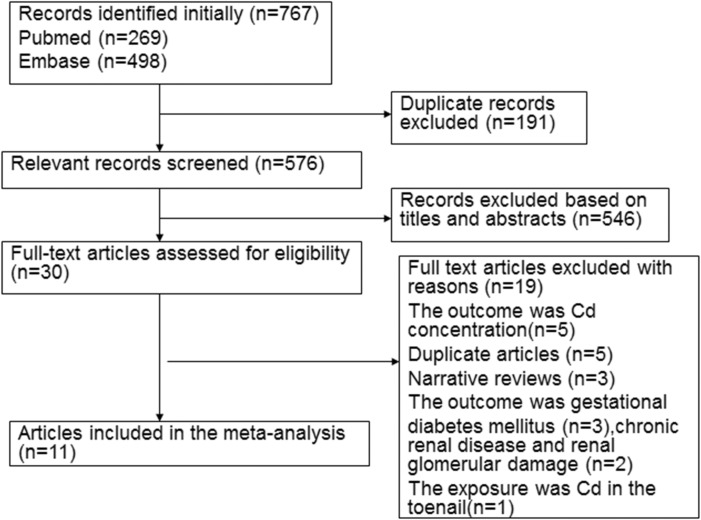
Flow diagram of the literature included

Following development of our search strategy, 767 records were initially identified. A total of 191 duplicate studies were excluded, 576 were subsequently screened, and 408 were excluded because titles and abstracts did not fit our criteria. Thirty full-text articles were reviewed for further assessment. Five articles were excluded because they were duplicate publications [14, 15, 22, 24, 28]; another five were excluded because Cd content was measured [33–37], three studies were excluded because the outcome was gestational diabetes mellitus [17, 18, 25], three were reviews [38–40], two were excluded because outcomes were related to chronic kidney disease [41] or renal glomerular damage [42] and one was excluded because the exposure was Cd in the toenail [13]. Finally, eleven studies met meta-analysis criteria and were included (Figure 1).

Table 1 summarizes individual characteristics of eleven included articles (two cohort studies [29, 30], and nine cross-sectional studies [8, 16, 19–21, 23, 26, 27, 31]). The articles were published from 2007 to 2017. Of the 11 studies, seven were published in Asia [16, 19, 21, 23, 26, 27, 31], another two were published in Europe [29, 30], one was published in North American [8] and one in Australia [20]. Numbers of DM patients ranged from 28 to 1,346 across all included studies. Seven studies used urinary Cd as biomarker for long-term Cd exposures [8, 16, 19, 20, 23, 27, 30], whereas five articles evaluated Cd exposure levels by estimating blood Cd [21, 26, 29–31]. The diabetes mellitus identification and adjusted covariates are shown in Table 2. Diabetes was defined based on self-reported physician diagnosis [8] and/or medication plus fasting plasma glucose [16, 19–21, 23, 26, 27, 30, 31], oral glucose tolerance test (OGTT) [30], or local diabetes register [29]. Most studies examined the association between Cd exposure and risk of DM [8, 19, 20, 23, 26, 27, 29], only three studies explored the relationship between Cd exposure and risk of type II DM [16, 30, 31]. Adjusted effect estimates were determined for most included studies, except for one [20]. Most risk estimates were adjusted for age (*n* = 9) [8, 16, 19, 21, 23, 26, 27, 29, 31], smoking status (*n* = 9) [8, 16, 21, 23, 26, 27, 29–31] and alcohol consumption (*n* = 6) [8, 16, 23, 26, 27, 31], Some studies were controlled for gender (*n* = 6) [8, 16, 19, 21, 26, 27, 31], body mass index (*n* = 4) [16, 19, 21, 23, 27] and waist circumference (*n* = 3) [8, 29, 30], but few were adjusted for ethnicity (*n* = 2)8,19, hypertension (*n* = 2) [23, 27] and physical activity (*n* = 2) [26, 27]. None of the studies were adjusted for exposure to other heavy metals (such as arsenic and bisphenol).

**Table 1:** Characteristics of cohort/cross-sectional studies included in the meta-analysis

Study	Year	Country	Study design	No. of cases	Sample size	Sex	Age, Median(Range), years	Exposure	Exposure assessment	Study period (years)
Haswell-Elkins	2007	Australia	A cross-sectional study	28	126	Female and male	36.2 (15–76)	U-Cd	An inductively coupled mass spectrometry	1996
Swaddiwudipong	2010	Thailand (Mae Sot District)	A cross-sectional study	348	5273	Female and male	52.8±11.9 (≥ 35)	U-Cd	A graphite tube atomic-absorption spectrometer.	2009
Barreqard	2013	Swede	A cross-sectional and prospective cohort study	68	2595	Female	NA (> 64)	U-Cd,B-Cd	An inductively coupled plasma-mass spectrometry	2001-2003
Moon	2013	Korea	A cross-sectional study (KNHANES)	333	F:1588; M:1596	Female and male	NA (≥30)	B-Cd	Graphite-furnace atomic absorption spectrometry and Zeeman background correction.	2009–2010
Borne	2014	Swede (Malmo¨ in southern Sweden)	A prospective cohort study (MDC)	622	4585	Female and male	NA (46-67)	B-Cd	An inductively coupled plasma mass spectrometry with an octopole reaction system.	1991–1996
Son	2015	Korea	A cross-sectional study (HESRAM)	158	719	Female and male	59.1 (40-70)	U-Cd	A flameless atomic adsorption spectrometry and graphite furnace attached to atomic absorption spectrophotometer.	2008–2011
Tangvarasittichai	2015	Thailand	A cross-sectional study	30	535	Female and male	NA (≥30)	U-Cd	A graphite tube atomic-absorption spectrometer.	2010–2011
Liu	2016	China	A cross-sectional study	102	1493	Female and male	DM:47 ± 6.7; Normoglycemia group: 41.8 ± 8.7	U-Cd	An inductively coupled plasma-mass spectrometry	2016
Menke	2016	USA	A cross-sectional study (NHANES)	1364	9447	Female and male	Diabetes: 58.6 ± 0.54; No diabetes: 45.6 ± 0.30	U-Cd	An inductively coupled plasma 8 mass spectrometry	1999–2010
Nie	2016	China	A cross-sectional SPECT-China study	565	5544	Female and male	NA (18-≥69)	B-Cd	A graphite furnace atomic absorption spectrometry.	2016
Li	2017	China	A cross-sectional study	122	559	Female and male	NA (40-92)	B-Cd	An inductively coupled plasma-mass spectrometry	2014-2016

B-Cd, blood cadmium; U-Cd, urinary cadmium; NA, not available.

**Table 2:** Diabetes mellitus identification, adjustment for covariates of cohort/cross-sectional studies included in the meta-analysis

Author	year	Diabetes mellitus identification	Adjustment for covariates
Haswell-Elkins	2007	Diabetes was defined as a fasting blood glucose level of 7.8 mmol/l or greater and/or a 2 h glucose test result over 11.0 mmol/l.	NA
Swaddiwudipong	2010	Diabetes was defined as fasting plasma glucose ≥ 126 mg/dl on 2 occasions or currently receiving anti-diabetic treatment.	Adjusted for age, alcohol consumption, tobacco smoking, body mass index, and hypertension.
Barreqard	2013	Diabetes was defined as FPG ≥ 6.1 (≥ 110) and/or 2 h post glucose load ≥ 11.1 mmol/l (≥ 200 mg/dl) measured at two occasions (OGTT).	Adjusted for by pack years of smoking, waist circumference, serum adiponectin.
Moon	2013	Diabetes was defined as fasting plasma glucose levels at 126 mg/dl, they were on diabetes treatment, or they reported a history of physician-diagnosed diabetes.	Adjusted for age, sex, region, smoking, alcohol consumption, and regular exercise.
Borne	2014	Incident cases of diabetes were identified from national and local diabetes registers.	Adjusted for age, waist circumference, and smoking status.
Son	2015	Diabetes was defined as the existence of past diabetes history, for cases in which treatment of diabetes resulted in measurement of more than 126 mg/dl in fasting blood glucose according to diabetes diagnostic criteria in American Diabetes Association	Adjusted for age (continuous), sex, ethnicity (non-Hispanic blacks, Mexican Americans, and others vs. non-Hispanic white), and BMI (continuous).
Tangvarasittichai	2015	Diabetes was defined as fasting glucose concentration of ≥ 126 mg/dl, non-fasting glucose concentration of ≥ 200 mg/dl, a self-reported physician diagnosis, or medication use.	Adjusted for diabetes, CKD, U-Protein/g CT, Cal/g CT, BMI, alcohol drinking, smoking, gender, age.
Liu	2016	Diabetes was diagnosed when FPG ≥ 7.0 mmol/L or use of anti-diabetic medications or as self-reported by physicians (the WorldHealth Organization guidelines).	Adjusted for gender, age, BMI, smoking status, drinking status, physical activity, education levels, urinary creatinine, hypertension, hyperlipidemia, and urinary PAHs level.
Menke	2016	Diabetes was defined as a self-reported previous diagnosis of diabetes or an HbA1c ≥ 6.5% (48 mmol/mol).	Adjusted for age, race, ethnicity, sex, menopausal status, education, income, smoking status, pack years smoked, alcohol consumption, waist circumference, C-reactive protein, high alanine aminotransferase, high gamma glutamyl transferase, daily calories consumed, percent of calories from saturated fat, and urinary creatinine.
Nie	2016	Diabetes was defined as a previous diagnosis by health care professionals or FPG ≥ 7.0 mmol/L.	Adjusted for age (continuous), sex, residence area, economic status, current smoker, hypertension, dyslipidemia, estimate glomerular filtration rate (continuous), blood lead (continuous), and BMI (continuous)
Li	2017	Diabetes was defined as random plasma glucose concentrations ≥ 11.1 mmol/L plus symptoms of diabetes, 2-hour post-load oral glucose tolerance test (OGTT) ≥ 11.1 mmol/L, or fasting plasma glucose (FPG) ≥ 7.0 mmol/L, an HbA1c ≥ 6.5%.	Adjusted for age, gender, BMI, family history, smoking and drinking status.

FPG, fasting plasma glucose; OGTT, oral glucose tolerance test; HbA1c, Hemoglobin A1c.

Included studies generally showed good methodological quality. Newcastle–Ottawa Scale (NOS) scores ranged from 5 to 7 (Table 3). Mean NOS score was 6.2.

**Table 3:** Quality assessment of eligible studies based on newcastle-ottawa scale

Author	year	Selection	Comparability	Exposure
Haswell-Elkins	2007	2	0	2
Swaddiwudipong	2010	3	2	1
Barreqard	2013	3	1	2
Moon	2013	3	2	2
Borne	2014	3	1	2
Son	2015	3	2	1
Tangvarasittichai	2015	3	2	2
Liu	2016	3	2	2
Menke	2016	3	2	2
Nie	2016	3	2	2
Li	2017	3	2	1

### U-Cd concentration and risk of DM

**Figure 2:**
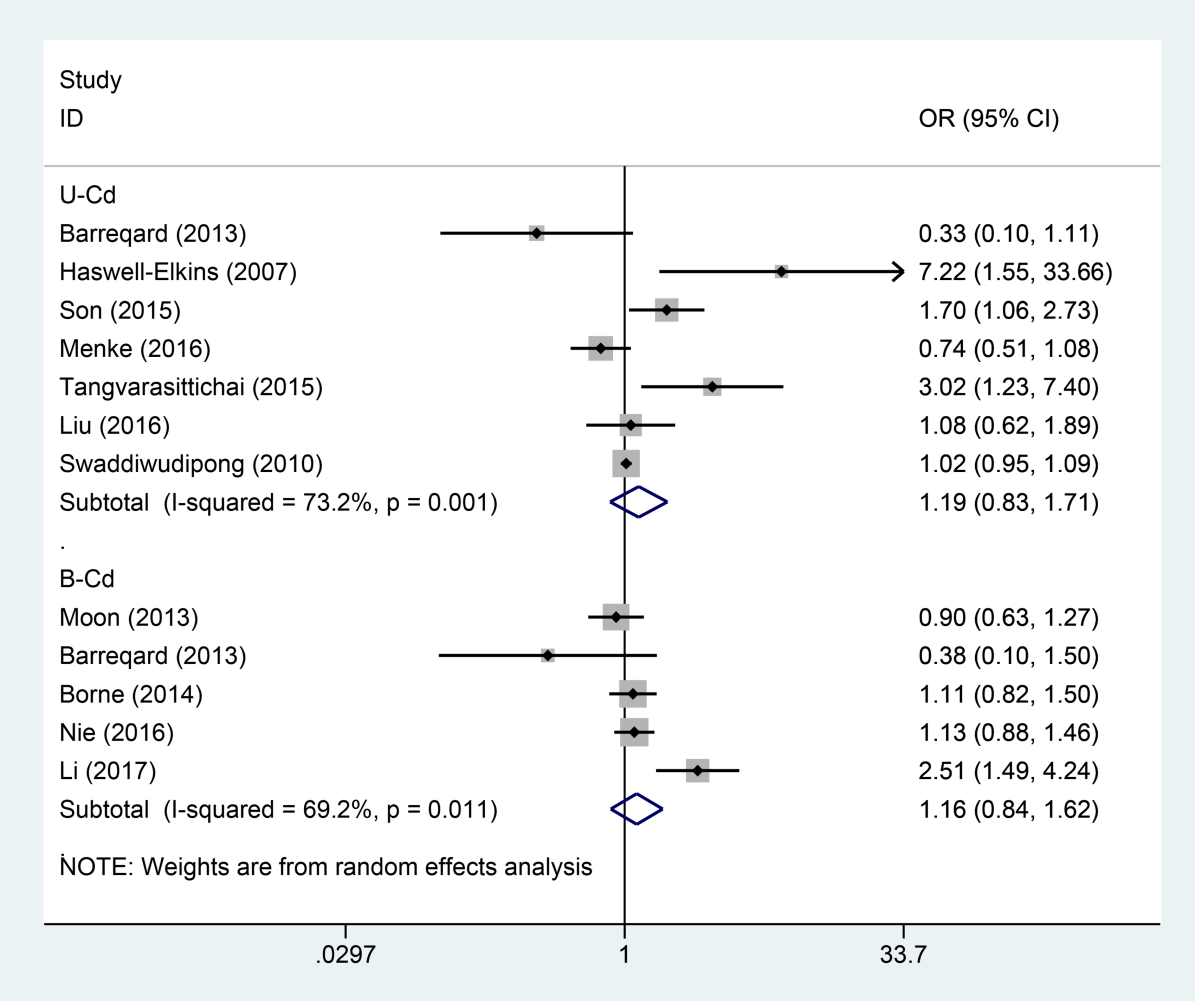
Forest plot for the association between U-Cd/B-Cd exposure and DM risk

**Figure 3:**
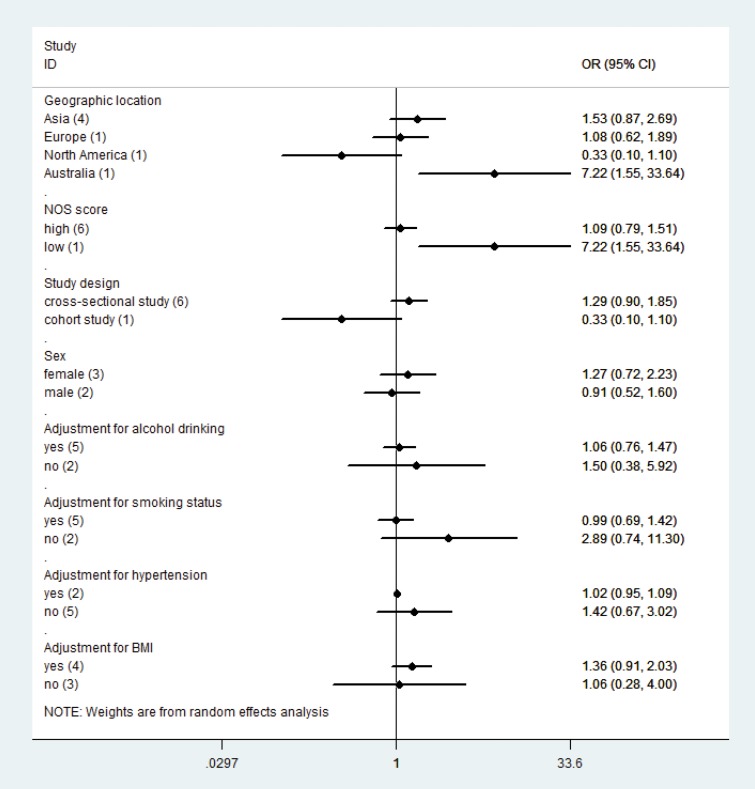
Subgroup analysis for U-Cd exposure and DM risk

Seven studies examined the association of U-Cd concentration with risk of DM. The overall OR was 1.19 times (odds ratio (OR) = 1.19; 95% confidence interval (CI = 0.83–1.71) for the highest category of Cd exposure compared with lowest category with significant heterogeneity (P_for heterogeneity_= 0.001, *I*^2^ = 73.2%) (Figure 2, Table 4). In sensitivity analysis, similar results were observed for DM risk; values ranged from 1.07 (95% CI = 0.76–1.52) with significant heterogeneity (*I*^2^ = 70.2%; P_for heterogeneity_= 0.005) (excluding the study by Tangvarasittichai et al. [16]) to 1.37 (95% CI = 0.87–2.17) with significant heterogeneity (*I*^2^ = 74.2%; P_for heterogeneity_= 0.002) (excluding the study by Menke et al. [8]). Subgroup analysis stratified by geographic region and gender, study design, NOS score, adjustment for covariates, exposure type, and similar results for DM (Figure 3, Table 4).

**Table 4:** Results of subgroup analysis between U-Cd concentration and risk of DM

	Studies, N	OR (95% CI)	*P*-value	*P* for heterogeneity	*I*^2^ (%)
Total	7	1.19 (0.83-1.71)	0.334	0.001	73.2
**Geographic location**					
Asia	4	1.53 (0.87-2.68)	0.142	0.007	79.7
North America	1	1.08 (0.62-1.89)	0.120	NA	NA
Europe	1	0.33 (0.10-1.11)	0.073	NA	NA
Australia	1	7.22 (1.55-33.66)	0.012	NA	NA
**NOS score**					
High	6	1.09 (0.79–1.51)	0.596	0.006	69.1
Low	1	7.22 (1.55–33.66)	0.012	NA	NA
**Study design**					
Cross-sectional study	6	1.29 (0.90-1.85)	0.160	0.002	73.7
Cohort study	1	0.33 (0.10-1.11)	0.073	NA	NA
**Gender**					
Female	3	1.27 (0.72-2.22)	0.747	0.157	46.0
Male	2	0.91 (0.52-1.60)	0.410	0.037	77.1
**Adjusted for confounders or important risk factors**					
**Alcohol drinking**					
yes	5	1.06 (0.76-1.47)	0.748	0.038	64.3
no	2	1.50 (0.38-5.91)	0.559	0.006	80.4
**Smoking status**					
yes	5	0.99 (0.69-1.41)	0.948	0.020	65.8
no	2	2.89 (0.74-11.32)	0.128	0.079	67.7
**Hypertension**					
yes	2	1.02 (0.95-1.09)	0.549	0.843	0.0
no	5	1.42 (0.67-3.03)	0.363	0.000	81.6
**BMI**					
yes	4	1.36 (0.91-2.02)	0.133	0.020	69.6
no	3	1.06 (0.28-3.99)	0.927	0.007	80.1

OR, odds risk; CI, confidence interval; High, NOS score of ≥ 6; Low, NOS score of < 6; NA, not available.

### B-Cd concentration and risk of DM

Five studies examined the association of B-Cd concentration with risk of DM. The summary OR was 1.16 times (odds ratio (OR) = 1.16; 95% confidence interval (CI = 0.84–1.62) for the highest category of Cd exposure compared with lowest category with low heterogeneity (P_for heterogeneity_= 0.011, *I*^2^ = 69.2%) (Figure 2). In sensitivity analysis, similar results were observed for DM risk; values ranged from 1.04 (95% CI = 0.87–1.25) (*I*^2^ = 9.7%; P_for heterogeneity_= 0.344) (excluding the study by Li [31]) to 1.26 (95% CI: 0.84–1.90) with significant heterogeneity (*I*^2^ = 72.1%; P_for heterogeneity_= 0.013) (excluding the study by Moon [26]). Subgroup analysis stratified by geographic region and study design, NOS score, adjustment for covariates, exposure type, and similar results for DM (Table 5).

**Table 5:** Results of subgroup analysis between B-Cd concentration and risk of DM

	Studies, *N*	OR (95% CI)	*P*-value	*P* for heterogeneity	*I*^2^ (%)
Total	5	1.16(0.84-1.62)	0.361	0.011	69.2
**Geographic location**					
Asia	3	1.30 (0.81-2.10)	0.274	0.006	80.8
Europe	2	0.81(0.31-2.11)	0.663	0.135	55.2
**NOS score**					
High	5	1.16(0.84-1.62)	0.361	0.011	69.2
Low	0	NA	NA	NA	NA
**Study design**					
Cross-sectional study	3	1.30 (0.81-2.10)	0.274	0.006	80.8
Cohort study	2	0.81(0.31-2.11)	0.663	0.135	55.2
**Adjusted for confounders or important risk factors**					
**Alcohol drinking**					
yes	2	1.47(0.54-4.03)	0.451	0.001	90.2
no	3	1.09(0.87-1.36)	0.441	0.310	14.7
**Smoking status**					
yes	5	1.16(0.84-1.62)	0.361	0.011	69.2
no	0	NA	NA	NA	NA
**Hypertension**					
yes	1	1.13(0.88-1.46)	0.344	NA	NA
no	4	1.16(0.70-1.91)	0.561	0.005	76.5
**BMI**					
yes	2	1.63(0.75-3.55)	0.221	0.007	86.1
no	3	0.97(0.72-1.29)	0.821	0.256	26.7

OR, odds risk; CI, confidence interval; High, NOS score of ≥ 6; Low, NOS score of < 6; NA, not available.

### Publication bias

**Figure 4:**
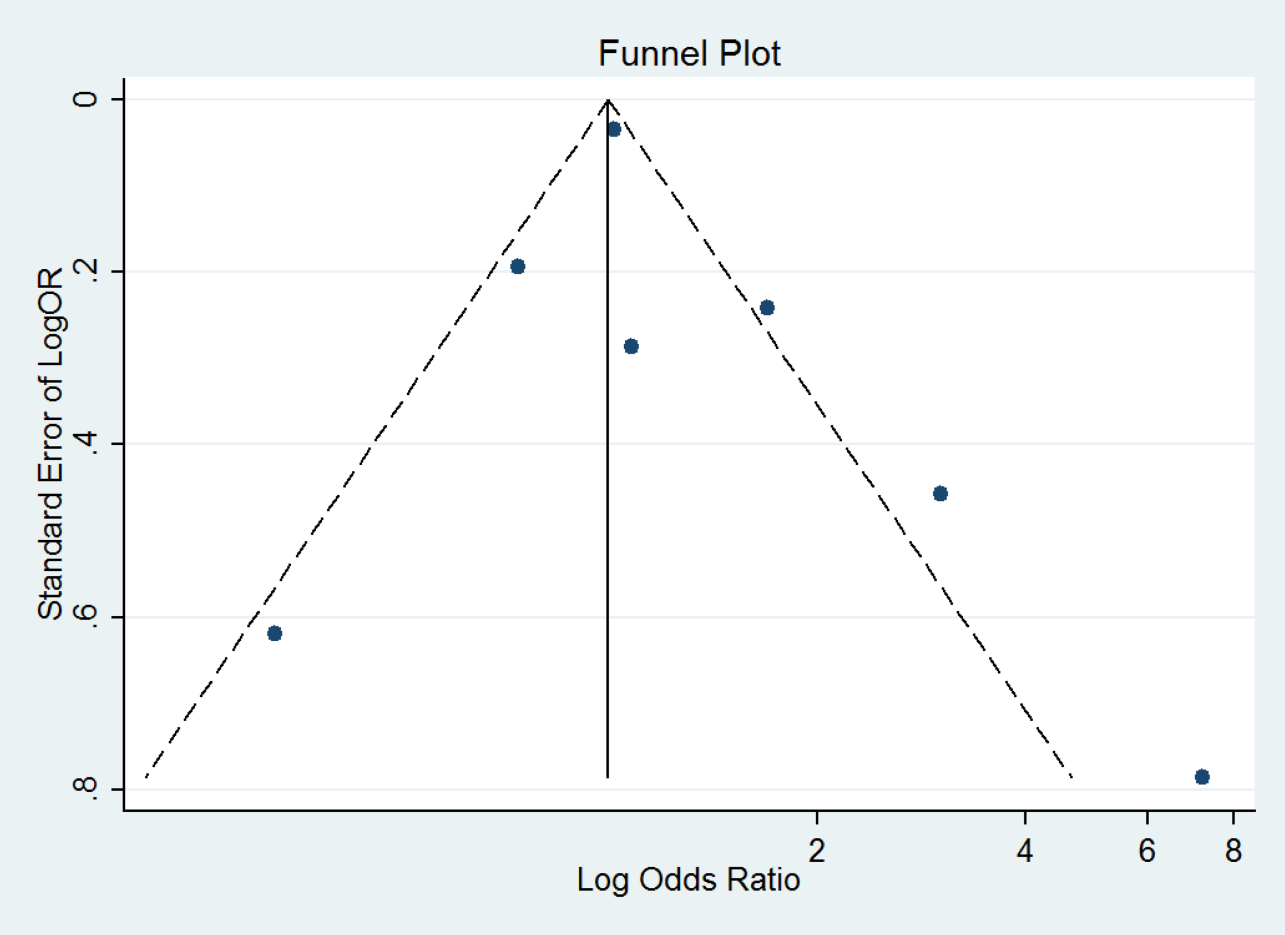
Funnel plot for publication bias analysis between U-Cd exposure and DM risk

Due to relatively limited number of eligible studies, we only conduct publication bias between U-Cd concentration and risk of DM, but not in B-Cd concentration. There was little evidence of publication bias with Egger funnel plot asymmetry (*P* = 0.368) and Begg rank correlation tests (*P* = 0.446). Funnel plot symmetry (Figure 4) was also examined.

## DISCUSSION

Cd is an ubiquitous industrial and naturally occurring environmental contaminant resulting from anthropogenic activity; it poses toxic effects on lungs, liver, testicles, kidneys, and bone tissues [11]. Recently, Cd exposure was associated with multiple adverse health effects, including osteoporosis and fractures [43, 44], renal dysfunction [45, 46], atherosclerotic plaques [47, 48], cancer [49, 50], and cardiovascular diseases [51, 52]. Cd is primarily considered a nephrotoxicant; however, numerous short- and long-term *in vivo* Cd exposure models showed that Cd can cause hyperglycemia and disrupt glucose homeostasis in experimental animals [39, 53]. Significant relationship was observed between Cd exposure and prevalence of prediabetes and/or T2DM8, [13–29]. Using *National Health and Nutrition Examination Survey* (NHANES) data, urine Cd was positively associated with diabetes [14, 15], but no correlation was detected in further studies [8]. In a cross-sectional study of Korean general population, B-Cd was associated with metabolic syndrome [26] but not diabetes [26]. In a case-control study in Pakistan, participants with diabetes exhibited higher levels of hair Cd than those without [54]. No statistically significant association was found between Cd exposure and diabetes in heavily Cd-contaminated area in Thailand [22–24]. However, in a study from the same group, diabetes prevalence significantly increased in subpopulation with continually high Cd exposure [55]. In three prospective studies, Cd exposure was unrelated to development of diabetes in two studies [29, 30] but correlation was observed in one study [13]. The most compelling evidence came from cross-sectional study by Menke et al. (2016), who used data from NHANES (1999–2010) to assess whether increased urinary cadmium (U-Cd) was associated with impaired T2D in the United States [8]. However, these studies included modest sample sizes, and magnitude of association varied among studies, with OR ranging from 0.74 (95% CI: 0.51–1.09) to 7.22 (95% CI: 1.52–33.04) and wide CI. Therefore, magnitude was limited by low precision of risk estimates. These epidemiological studies showed absence of comprehensive assessment in Cd exposure. Therefore, we conducted comprehensive meta-analysis to investigate the association between Cd exposure and DM risk.

In this meta-analysis, we analyzed associations between urinary/blood Cd exposure and DM risk. The present study is the first to provide comprehensive insights into effects of Cd exposure and DM-associated risks. Using random-effects model, 10 observational studies were included, and overall results proved that high Cd exposure is not associated with increased DM risk among the general population.

Results from subgroup analyses indicated potential sources of heterogeneity; these sources include geographic region, study design, NOS scale quality, gender, adjusted confounders or important risk factors, and method of Cd assessment. Despite intrinsic limitations of observational studies, subgroup analysis provided notable results. When stratified by gender, association remained nonsignificant for both male and female. Cd concentrations in urine and whole blood are the most common biomarkers for Cd exposure. Therefore, in kidneys, urinary Cd mainly reflects Cd accumulation, which is determined by long-term exposure, whereas whole blood Cd demonstrates combined current and historical exposures. In subgroup analysis results, which were stratified using Cd exposure assessment, both urinary and blood Cd are not associated with increased DM risk. In epidemiological studies, the most common biomarker for Cd exposure may be urinary Cd. However, using urinary or blood Cd levels limits measurement of Cd exposure. Internal or absorbed dose of Cd in renal cortex is higher than that of blood or urine Cd levels in measuring low-level and long-term Cd exposure, because Cd concentration in renal cortex accurately represents lifelong Cd exposure [56, 57]. However, none of considered studies measured Cd concentration in renal cortex in meta-analysis. Smoking tobacco cigarettes is a major potential source of Cd exposure in the general population, and DM risk is increased among smokers. Therefore, we performed subgroup analyses adjusted for smoking status in a multivariable model to minimize possible non-Cd mediated negative effects of tobacco smoking on blood glucose. Only five publications [8, 16, 24, 27, 30] were adjusted for smoking status, and results showed that Cd exposure is not related to increased DM risk (Table 2).

Development of DM represents interaction of environmental exposures, lifestyle factors, and genetic predisposition. Pathogenesis of both diseases can be considered a continuum of dysglycemia with development of impaired insulin secretion and insulin resistance as common pathogenic link. Human tissue sample studies indicated that Cd may preferentially accumulate in pancreatic islets [58]. Mechanism of Cd-induced diabetes remains uncertain but possibly involves damage to insulin-producing β-cells in islets of Langerhans; in pancreas of Cd-exposed rats, such cells secrete substantially less insulin than unexposed ones [38, 39, 59]. In cultured rat pancreatic β-cells, Cd increased reactive oxygen species, induced oxidative stress, and catalyzed cell death [60]. Alternatively, either urinary excretion of Cd or body burden of Cd in humans may be increased by diabetes-related changes in renal function or other pathophysiological aspects of impaired glucose tolerance (IGT).

The present meta-analysis exhibited several strengths. First, this study was the first to investigate association between Cd exposure and DM risk. Second, large sample size improved risk estimate accuracy and resulted in well-founded conclusions based on meta-analysis. Pooled estimates were robust across sensitivity and subgroup analyses, and publication bias was not detected. Conclusions from combined estimates were more reliable than from single studies because overall OR was based on large sample size and exhibited sufficient power.

Nevertheless, some limitations should be considered in the present meta-analysis. First, almost all related previous studies were cross-sectional or retrospective, except for four prospective studies. Cross-sectional and case-control studies have inherent limitations, such as selective bias and recall or memory bias. Therefore, large prospective studies are needed to confirm such findings. Second, evidence from epidemiological studies indicated that Cd may exacerbate harmful renal effects of diabetes. However, owing to numerous other confounding environmental factors inherent in these epidemiological studies, difficulty arises from establishing cause-and-effect relationships. For example, increased probability of individuals becoming diabetic can be attributed to other environmental toxins, such as arsenic [61, 62] and bisphenol A [25, 63], or generalized inflammation and/or oxidative stress response [64]. Additional epidemiological studies are needed to rule out effects of such confounding variables on possible link between Cd and diabetes. Meanwhile, experimental study results in animals provided direct evidence for such a link [65, 66]. Third, independent measurement errors were possibly present in most included studies because multiple metals in several included studies were examined in same urine/blood samples using same assays; results then were potentially misleading. Moreover, we cannot exclude possibility of false positives. Therefore, associations observed in meta-analysis should be further investigated in future studies. Fourth, although the similar feature of the included studies as the uniform feature was observed, the present meta-analysis lacks information on type of diabetes, and we could not differentiate type 1 from type 2 DM in most included studies. We were also concerned that errors inevitably increase during DM diagnosis. Imprecise diagnosis of DM possibly attenuated true associations. Fifth, during follow-up, blood and urinary Cd levels probably declined. Therefore, in the present study, tentative Cd levels were possibly too low to have caused diabetogenic effects. Sixth, between-study heterogeneity is common in the meta-analysis, and it is essential to explore the potential sources of between-study heterogeneity. We also performed sensitivity analyses and subgroup analyses to determine the sources of heterogeneity, but heterogeneity was still observed. The potential for misclassification of exposure to cadmium and type 1/2 DM, Imprecise diagnosis of DM may contribute to the heterogeneity for all studies in the summary analysis.

In summary, the meta-analysis suggests that high Cd exposure may not be risk factor for DM in the general population. Further prospective studies should be conducted to determine possible association between low-level Cd exposure of general populations and increased risk of DM.

## MATERIALS AND METHODS

### Data source and search strategy

Literature search was performed in April 2017 without any limitations. Primary sources were electronic databases of PubMed and EMBASE. To identify eligible studies, main searches employed various combinations of Medical Subject Headings (MeSH) and non-MeSH terms: “diabetes mellitus” OR “diabetes” combined with “cadmium”. Reference lists of all studies and published systematic reviews and meta-analyses were screened to identify other potentially eligible studies. Main search was completed independently by investigators. Discrepancies were solved by consulting a researcher who was not involved in initial procedures.

### Eligibility criteria and study selection

Studies were considered eligible for inclusion in meta-analysis when they met the following criteria: (1) exposure of interest was urinary/blood Cd; (2) outcome of interest was DM risk; (3) study design was cohort, case control, or cross-sectional study; (4) RR, OR, or hazard risk (HR) with corresponding 95% CI were reported or provided sufficient data to estimate crude OR, RR, or HR values with corresponding 95% CI. When included population was duplicated in more than one study, only the study with most comprehensive information was included.

### Data extraction and quality assessment

Two reviewers extracted data independently using predefined data extraction form. Data included the following: first author, publication year, study design, country, total number of cases and subjects, sex, Cd exposure type, and diagnosis criteria for DM and adjusted variables. Adjusted OR was extracted as preference over non-adjusted OR. Unadjusted OR and CI were calculated whenever OR was not provided. When more than one adjusted OR was reported, the ratio with most number of adjusted variables was selected. Disagreements between authors (JKS and XHY) were resolved through discussion and consensus.

Methodological quality of included studies was assessed using NOS [67], which consists of three factors: patient selection, comparability of study groups, and assessment of outcomes. A score of 0–9 (allocated as stars) was allocated to each study. Observational studies achieving six or more stars were considered to be of high quality.

### Statistical analysis

We used OR with 95% CI as common measure across all studies. Cd-caused DM is a rare event. Therefore, in the cohort study, RR and HR were considered OR approximations. Two articles did not report overall risk estimates but instead separately presented results for men and women [19, 23]. We combined results using fixed effects and included pooled risk estimates in primary analysis. In one study, OR failed to provide reliable results; therefore, we computed crude risk estimates and corresponding CI20. Aggregated results and 95% CIs for effect sizes were calculated using inverse-variance weighted random-effect meta-analysis, which incorporated both within- and between-study variabilities [68]. *I*^2^ was used to assess heterogeneity across studies, with *I*^2^ values of 0%, 25%, 50%, and 75% representing no, low, moderate, and high heterogeneities, respectively. Sensitivity analysis was conducted to evaluate data robustness and stability by sequentially omitting one study on each turn. Subgroup analysis was stratified using study design, geographic region, gender, exposure type, adjustment for covariates, and NOS quality.

Consistent with publication bias, small study bias was evaluated using statistical tests (Begg rank correlation test [69] and Egger's linear regression test [70]) and visual examination of funnel plot of each trial effect size against standard error. Results indicated publication bias when *P* < 0.10. All statistical analyses were conducted using Stata version 13.1 (Stata Corp, College Station, TX, USA).
